# Supplementary material for: Identification and functional analysis of non-coding regulatory small RNA FenSr3 in Bacillus amyloliquefaciens LPB-18
Source: PeerJ. 2023 May 15;11:e15236. doi: 10.7717/peerj.15236 (PMC10194069; doi:10.7717/peerj.15236)
Supplement: Supplemental Information 4 [file peerj-11-15236-s004.zip › KO/CK-vs-T1_map/map02010.html]

KEGG PATHWAY: ABC transporters - Reference pathway


|  |  |
| --- | --- |
| **ABC transporters - Reference pathway** |  |

[
Pathway menu
| Organism menu
| Pathway entry
| Show description
| User data mapping
]

The ATP-binding cassette (ABC) transporters form one of the largest known protein families, and are widespread in bacteria, archaea, and eukaryotes. They couple ATP hydrolysis to active transport of a wide variety of substrates such as ions, sugars, lipids, sterols, peptides, proteins, and drugs. The structure of a prokaryotic ABC transporter usually consists of three components; typically two integral membrane proteins each having six transmembrane segments, two peripheral proteins that bind and hydrolyze ATP, and a periplasmic (or lipoprotein) substrate-binding protein. Many of the genes for the three components form operons as in fact observed in many bacterial and archaeal genomes. On the other hand, in a typical eukaryotic ABC transporter, the membrane spanning protein and the ATP-binding protein are fused, forming a multi-domain protein with the membrane-spanning domain (MSD) and the nucleotide-binding domain (NBD).

|  |  |  |
| --- | --- | --- |
| Reference pathway | 184% 150% 122% 100% 82% 67% 55% | 图片下载 |
